# A SMARC Effect for Loudness

**DOI:** 10.1177/2041669517742175

**Published:** 2017-11-21

**Authors:** Elena Bruzzi, Francesca Talamini, Konstantinos Priftis, Massimo Grassi

**Affiliations:** 154917 Department of General Psychology, University of Padova, Italy

**Keywords:** audition, cognition, spatial selection/modulation, SMARC effect, smarc effect, music

## Abstract

Various reports suggest that the pitch height of musical tones may be represented along a mental space, with lower pitch heights represented on the left or lower sectors and higher pitch heights represented on the right or upper sectors of the mental space. Given that in Western languages the loudness of tones is often addressed spatially, with loud sounds referred to as “high” and quiet sounds referred to as “low,” here we investigated whether loudness might also have a spatial representation. Participants judged whether a tone was louder or quieter than a reference tone, by pressing two keys: one at the top and the other at the bottom of a response box. Participants were faster in a situation where they pressed the key at the top to report louder sounds, and the key at the bottom to report quieter sounds, than vice versa. This result supports the view that loudness, like other types of magnitudes, might be represented spatially.

Quantities and magnitudes might be mentally represented along a spatial continuum (Brugger, 2008). A classic example is that of numbers. It seems that numbers are spatially represented with small numbers associated with the left side of a mental number line and large numbers with its right side. Therefore, in speeded tasks, participants respond faster to small numbers when responses are left sided and respond faster to large numbers when responses are right sided (the SNARC effect [Spatial–Numerical Association of Response Codes]; Dehaene, Bossini, & Giraux, 1993). A distance effect in number processing has also been reported (Moyer & Landauer, 1967): That is, the time required to process a number varies inversely as a function of the distance between the number and a reference number (i.e., the larger the distance, the faster the reaction time [RT]).

The origin of spatial–numerical associations is not clear. The results on preverbal children and new-born chicks suggest that spatial–numerical associations are automatic and innate (Bulf, de Hevia, & Macchi Cassia, 2015; Rugani, Vallortigara, Priftis, & Regolin, 2015). However, other studies suggest that spatial–numerical associations might be modulated by linguistic habits, such as the direction of reading and writing (Shaki, Fischer, & Petrusic, 2009) or by training (Lidji, Kolinsky, Lochy, & Morais, 2007).

Pitch height of musical tones might be spatially represented too. This effect has been termed the SMARC effect (Spatial–Musical Association of Response Codes; Cho, Bae, & Proctor, 2012; Lidji et al., 2007; Pitteri, Marchetti, Priftis, & Grassi, 2017; Rusconi, Kwan, Giordano, Umiltà, & Butterworth, 2006). For example, when asked to judge whether a tone was amplitude modulated or not (i.e., vibrato), participants were faster when the pitch of the tone was high and the response was given by pressing a button that was placed at the top of a vertically oriented response box. In contrast, participants were faster when the pitch of the tone was low and the response was given by pressing a button at the bottom of a vertically oriented response box (Pitteri et al., 2017).

The SMARC effect has been reported, both for musicians and nonmusicians, when response keys are placed radially (near-far; e.g., Rusconi et al., 2006), and for nonmusicians, when response keys are positioned vertically (e.g., Pitteri et al., 2017). In contrast, only musicians show the SMARC effect when response keys are placed horizontally (i.e., left–right) and the pitch is task irrelevant, whereas nonmusicians show it only when the pitch is task relevant (e.g., Cho et al., 2012; Lidji et al., 2007). Noticeably, the space–pitch relation has been observed also in nonspeeded tasks (Hartmann, 2017) and in nonspeeded tasks with young participants. However, although the space–pitch association seems clear in 4- or 5-year-old children (Nava, Grassi, & Turati, 2016), there is still debate as to whether it can be observed preverbally in newborns (Lewkowicz & Minar, 2014; Walker et al., 2010).

The SMARC effect has been widely investigated for pitch. Nonetheless, musical tones consist of (at least) two other dimensions beyond pitch height: timbre and loudness. In many Western languages, the loudness of sounds is conveyed “spatially”: Loud sounds are “high,” whereas quiet sounds are “low.” However, in comparison to pitch, little is known about whether loudness might also be spatially represented and, if yes, how this representation might influence response selection. The possible spatial representation of loudness (i.e., a SMARC effect for loudness) has been investigated in three studies (i.e., Chang & Cho, 2015; Hartmann & Mast, 2017; Fernandez-Prieto, Spence, Pons, & Navarra, 2017). Hartmann and Mast (2017, Experiment 2c) presented recordings of single digits at a high (or low) intensity and asked participants to respond with a left or right keyboard key whether the digit was quiet or loud. The authors observed faster reactions to quiet digits when the response was executed with a left-sided key and faster responses to loud digits when the response was executed with the right-sided key. In Chang and Cho (2015), participants listened to a reference tone, which was fixed in frequency (1,000 Hz) and in intensity. The reference tone was followed by a probe tone, which had the same frequency as that of the reference tone, but could be either quieter or louder than the reference tone. Participants judged whether the probe tone was quieter or louder than the reference tone, by pressing either the left-sided or the right-sided response key of a horizontally oriented response box. The results were mixed, revealing a SMARC effect for loudness only for the right side (i.e., faster responses to high-intensity tones, than to low-intensity tones, when responses were executed on the right). In contrast, RTs were similar for low- and high-intensity tones when responses were executed on the left.

The results of Hartmann and Mast (2017) and Chang and Cho (2015) suggest a possible left–right spatial representation of loudness with quiet sounds represented on the left and loud sounds represented on the right. However, as mentioned earlier, loudness is often addressed along the vertical dimension: high and low. Fernandez-Prieto et al. (2017) asked participants to respond to the loudness of sounds with the response buttons placed along the vertical axis (see Fernandez-Prieto et al., 2017, Figure 3). Similarly to Chang and Cho (2015), Fernandez-Prieto et al. asked the participants to judge whether the second of two consecutive tones with fixed frequency (261 Hz) was louder than the first. The authors observed faster and more accurate responses to loud sounds when the response was executed with the top button and faster and more accurate responses to quiet sounds when the response was executed with the bottom button. Chang and Cho (2017) and Fernandez-Prieto et al. (2017) used no silent interval to separate the two tones presented on each trial. Therefore, the first tone masked the audibility of the beginning of the second tone (i.e., forward masking), in particular in those trials where the second tone was quieter than the first (Moore, 2013). This introduced an uncontrollable factor in the speeded motor response. In addition, all previous studies investigating loudness tested relatively small samples of participants (i.e., *N* < 27 participants).

In the present study, like in Fernandez-Prieto et al. (2017), we investigated whether the SMARC effect for loudness can be observed on the vertical dimension, by means of a well-powered experimental design. Participants were asked to judge whether a probe tone was louder or quieter than a reference tone, by pressing one of two keys of a vertically oriented response box. On one hand, we expected faster and more accurate responses when the probe tone was louder than the reference and the response was executed with the upper button of the response box. On the other hand, we expected faster and more accurate responses when the probe tone was quieter than the reference tone and the response was executed with the lower button of the response box. Differently from Fernandez-Prieto et al. (2017), here the two tones composing each trial were separated by a 500-ms long silent interval to prevent forward masking.

## Method

### Participants

Forty-four nonmusicians, all students at the University of Padova, volunteered in the experiment (19 males, mean age = 23.4 years, *SD* = 2.58 years). The number of participants was selected a priori by means of the program G*Power 3 (Faul, Erdfelder, Lang, & Buchner, 2007) and by setting the statistical power of the experiment to 95%. All were Italian, and all but three were right handed. None of the participants reported taking music lessons (besides the compulsory music classes in the primary and middle school, which consisted in 2 hr a week until the average age of 13 years). All had normal hearing with left–right audiometric thresholds at frequencies 500, 1000, and 4000 below 30 dB HL.

### Apparatus, stimuli, and procedure

Participants were tested individually in a single-walled IAC soundproof booth. The experiment was administered by means of an ASUS computer connected to a monitor NEC MultiSync FE950+ and to an M-AUDIO FastTrack Pro sound card. The output of the sound card was delivered to a pair of Sennheiser HD 580 headphones. Sounds and experiment were programmed in Matlab with freely downloadable Matlab toolboxes (Kleiner et al., 2007; Soranzo & Grassi, 2014).

**Figure 1.**
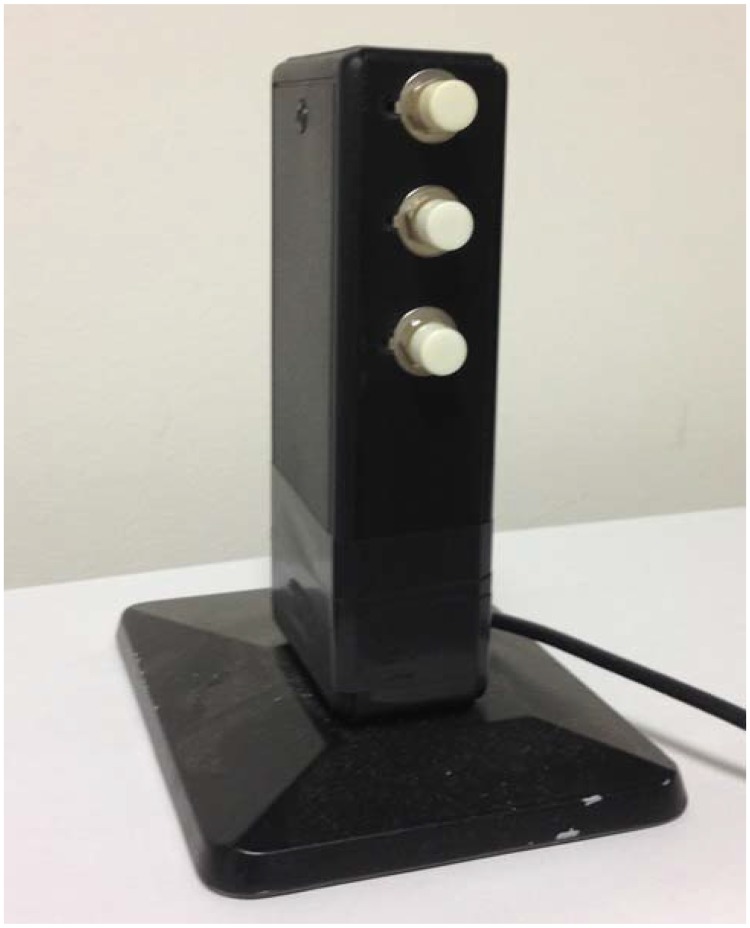
Photo of the custom-made response box used in the experiment.

On each trial, participants were presented with one complex tone (the reference tone) and one probe tone of identical pitch, timbre, and duration. The tone was 150 ms long and consisted of five harmonics, with a fundamental frequency of 550 Hz. The reference tone was presented at 70 dB SPL and it was followed by the probe tone. The probe tone could have either a lower intensity than that of the reference tone (−6, −12, or −18 dB) or a higher intensity than that of the reference tone (+6, +12, or +18 dB). Reference and probe tones were separated by a silent interstimulus interval of 500 ms to avoid forward masking (Moore, 2013). On each trial, participants judged whether the probe tone was louder or quieter than the reference tone, by pressing either of two keys: one key for “louder” tones and the other key for “quieter” tones. The two keys were placed on a custom-made, vertically oriented response box. The distance between the two keys was 7 cm (see Figure 1).

Participants used their thumbs to press the keys. Half of the participants used their right thumb for the top key and the left thumb for the bottom key. The other half of the participants used the opposite pattern. The top key was called the “rough key,”^1^ whereas the bottom key was called the “smooth” key, to avoid any explicit reference to the words “high” and “low” while giving the instructions to the participants.

The experiment was divided into two sessions. The order of the two sessions was counterbalanced between participants. In the first session, participants pressed the top key when the probe tone was louder than the reference tone and the bottom key when the probe tone was quieter than the reference tone (compatible condition). In the second session, participants pressed the top key when the probe tone was quieter than the reference tone and the bottom key when the probe tone was louder than the reference tone (incompatible condition). Participants were instructed to respond as quickly and as accurately as possible.

Participants had 2,850 ms to respond after the onset of the probe tone. Each session was divided into three blocks of 60 trials each. The duration of each session was about 8 min. While performing the experiment, participants were blindfolded, to avoid any interference from visual stimuli on tone processing. Participants were encouraged to take short breaks between blocks. Before the experiment, participants were given a training block of 10 trials, during which a visual feedback was provided (green dot for correct answers and red dot for incorrect answers). Within each block, the trials were presented in random order. The whole experiment lasted about 20 min.

### Design

The independent variables were Compatibility (two levels: compatible vs. incompatible) and Distance (six levels: −18, −12, −6, +6, +12, and +18). The dependent variables were RTs and accuracy.

## Results

**Figure 2.**
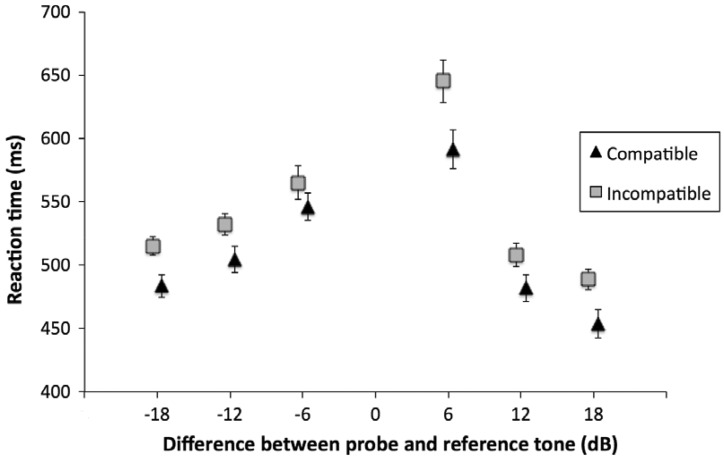
Mean reaction times as a function of the intensity difference between probe and reference tone. Triangles represent the compatible trials, whereas squares represent incompatible trials. Error bars represent confidence intervals (95%), calculated by means of the Cousineau–Morey correction for within-participant designs (O’Brien & Cousineau, 2014).

Anticipations (i.e., RTs occurring before the onset of the probe tone), out of time responses (i.e., RTs longer than 2,850 ms), and incorrect responses were excluded from the analysis (7.5% of the data). Of the remaining data, RTs longer or shorter than two standard deviations from the mean of each participant were also excluded. Finally, the mean RT of each participant for intensity level of the probe tone and response side (i.e., Compatibility: compatible vs. incompatible) was calculated. A two-way analysis of variance for repeated measures was performed on RTs, and corrected for a violation of sphericity (Greenhouse–Geisser correction). The main effect of Compatibility was significant (Figure 2): *F*(1, 43) = 5.63, *p* = .022, $\eta_{p}^{2}$*^ ^= *.12: RTs to compatible trials (*M* = 510 ms, *SD* = 132 ms) were faster than RTs to incompatible trials (*M* = 542 ms, *SD* = 167 ms). The main effect of Distance was also significant, *F*(5, 215) = 50.9, *p* < .001, $\eta_{p}^{2}$*^ ^= *.54: the larger the distance, in intensity, between the reference and the probe tone, the faster the response. We further investigated the effect of distance by running tests of polynomial contrasts. Specifically, the quadratic contrast was significant *t* = 6.85, *p* < .001. Finally, the interaction between Distance and Compatibility was not significant, *F*(5, 215) = 1.30, *p* = .263, $\eta_{p}^{2}$*^ ^= *.03. RTs are represented in Figure 2.

**Figure 3.**
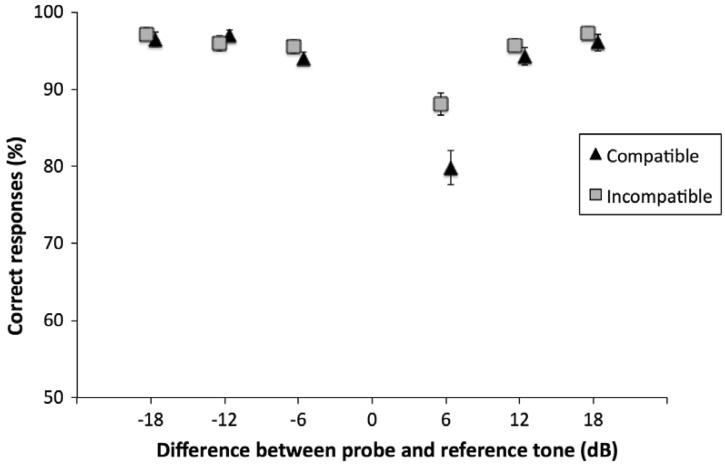
Proportion of correct responses as a function of the intensity difference between probe and reference tone. Triangles represent compatible trials, whereas squares represent incompatible trials. Error bars represent confidence intervals (95%), calculated by means of the Cousineau–Morey correction for within-participant designs (O’Brien & Cousineau, 2014).

A two-way analysis of variance for repeated measures was calculated on accuracy and corrected for a violation of sphericity (Greenhouse–Geisser correction). The main effect of Compatibility was not significant, *F*(1, 43) = 2.87, *p* = .098, $\eta_{p}^{2}$^ ^= .06. In contrast, the main effect of Distance was significant, *F*(5, 215) = 38.96, *p* < .001, $\eta_{p}^{2}$^ ^= .47, indicating that accuracy increased as a function of the distance between the probe and the reference tone. Again, to further investigate the Distance effect, we ran polynomial contrasts tests, and the quadratic contrast for the distance was significant, *t* = − 7.34, *p* = <.001. Finally, there was a significant Compatibility by Distance interaction, *F*(5, 215) = 7.61, *p* < .001, $\eta_{p}^{2}$^ ^= .15, suggesting that particular combinations of probe level and Compatibility led to more mistakes than other combinations did. In particular, performance was almost at ceiling for all probe intensities except when the probe was more intense than the reference of 6 dB. Accuracies are represented in Figure 3.

## Discussion

The aim of the present study was to investigate whether loudness might have a vertical mental spatial representation. In many Western languages, loudness is conveyed by means of spatial terms (e.g., loud sounds are “high” and quiet sounds are “low”). In addition, evidence from some recent studies supports the view that loudness, as well as pitch, might be spatially represented (Chang & Cho, 2015; Fernandez-Prieto et al., 2017; Hartmann & Mast, 2017). Therefore, here we expected that the variation of the probe intensity, with respect to a reference tone, would lead to higher performance (shorter RTs and fewer errors) in case of spatial compatibility between stimulus loudness (low vs. high) and response position (low placed vs. high placed). Results showed that responses to compatible trials were faster than responses to incompatible ones, suggesting the existence of a SMARC effect for loudness (from now on, the loudness-SMARC effect). Furthermore, RTs to loudness levels at the largest distances from the reference (−18 dB and +18 dB) were faster, than responses to the closer levels (−6 dB and +6 dB). Thus, a distance effect was present (Moyer & Landauer, 1967): That is, the time required to process a stimulus varied inversely as a function of the probe–reference distance on the judged dimension (i.e., the larger the distance, the faster the RT). This effect might be due to the difficulty of the task: Although discriminations were almost at ceiling for all stimuli pairs, probe intensities that were more distant from the reference were somewhat easier to discriminate, thus eliciting faster responses.

No significant interaction between Compatibility (compatible vs. incompatible trials) and the loudness level was found, meaning that the difference between RTs in the compatible condition and in the incompatible condition did not vary proportionally as a function of the distance between the probe and the reference tone. This result is different from that observed for the SMARC effect for pitch, in which the difference of RTs between the compatible and the incompatible condition is usually proportionally smaller for pitches close to the reference and larger for the largest distance, suggesting that individual pitches are aligned along a mental line. This might be explained by the fact that loudness is a more coarse perceptual continuum in comparison to pitch; that is, although our ability to discriminate loudness is high (∼1 dB; see Kidd, Watson, & Gygi, 2007), the way we categorize loudness is rather coarse. For example, in the Western musical notation, loudness has only a few categories (i.e., six: from “pianissimo” to “fortissimo”), whereas the notation of pitch divides octaves into 12 chroma (Oxenham, 2012).

The pitch of a tone (i.e., its frequency) and its loudness might be processed in different ways: Processing of loudness resembles more that of the magnitude of numbers, than that of pitch, as if loudness were perceived as a “magnitude.” This distinction reminds of a classic psychophysical distinction between metathetic and prothetic continua with pitch belonging to the former and loudness to the latter of the two types of continua (Stevens, 1957). Interestingly, the results of a study by Hartmann and Mast (2017), regarding the association between numbers and loudness processing, revealed that the two processes are not independent and they influence each other. This evidence is in line with the hypothesis that there is a part of the cognitive system, which is dedicated to the processing of the magnitude of stimuli irrespectively of their nature (see also Brugger, 2008). This evidence is also in line with ATOM (A Theory Of Magnitude; Walsh, 2003), which suggests that the magnitude of stimuli of different nature (numbers, space, and time) is processed within a generalized “magnitude network.” It is worth noticing, however, that the participants in our study were Italian speakers. Thus, the loudness-SMARC effect found here might be due to the verbal labels that are used in Western languages to refer to loud and quiet sounds: respectively, “high” and “low.” This linguistic habit, together with the binary response adopted in the current study, could be responsible for the results observed here. In other words, stimuli and response alternatives might be coded coherently (i.e., polarity correspondence), and this coherence might be sufficient to observe the loudness-SMARC effect (Proctor & Cho, 2006; but see Di Rosa et al., 2017; Dollman & Levine, 2016). Further research is needed to disentangle the influence of linguistic habits on loudness processing as well as that of the experimental method.

The analysis of response accuracy showed that participants were equally accurate on compatible and incompatible trials. Accuracy was at ceiling for all the probe–reference pairs except when the probe was 6 dB higher than the reference. The significant interaction observed in the statistical analysis was boosted by this particular data point. It is not clear why the accuracy dropped for this stimulus pair. Loudness differences between probe and reference tone were selected in such a way to exceed the discrimination threshold for intensity (i.e., less than 1 dB; see Kidd et al., 2007). One possible explanation for the drop in accuracy at this particular loudness difference and, in particular, only when the probe was louder than the reference might be due to a fast adaptation to the loudness of the reference tone that attenuates the loudness of the successive probe.

The results of the current experiment extend those of Chang and Cho (2015) and Hartmann and Mast (2017), who found evidence for an asymmetric spatial association of loudness on the horizontal dimension. Here, the spatial mapping of loudness was observed for the vertical plane and for both its sides (upper vs. lower) such as in the study by Fernandez-Prieto et al. (2017). Noticeably, our results were obtained with a larger statistical power than that offered in these few available previous demonstrations. In addition, here the SMARC effect for loudness along the vertical dimension was observed in a situation avoiding the possible (and unpredictable) role of forward masking because the two sounds composing each trial were separated by a silent interval of 500 ms. In conclusion, the current results seem to suggest that loudness, like other types of magnitude, can be represented spatially, although alternative explanations (notably, polarity correspondence; Proctor & Cho, 2006) remain to be explored. In addition, another factor not considered here but that should be taken into account by future research is the level of musical expertise of the participants. Musicians participated in some of the previous studies investigating the SMARC effect for loudness (Chang & Cho, 2015; Fernandez-Prieto et al., 2017). However, Chang and Cho (2015) did not include musical expertise in the statistical analyses, and Fernandez-Prieto et al. (2017) did not report the number of musicians and nonmusicians participating in their study, although the latter do report that musical expertise did not modulate their findings. Since musical expertise does seem to modulate the SMARC effect for pitch (e.g., Lega, Cattaneo, Merabet, Vecchi, & Cucchi, 2014; Rusconi et al., 2006; Stewart, Verdonschot, Nasralla, & Lanipekun, 2013; Timmers & Li, 2016), this factor clearly warrants further study for the SMARC effect for loudness demonstrated here.

## Note

1 The top key was covered with paper sellotape so that it could be recognized at touch.
